# Osteopathic Manipulative Treatment for a Chronic Rotator Cuff Tear: A Case Report

**DOI:** 10.7759/cureus.46292

**Published:** 2023-09-30

**Authors:** Luke J Scypinski, Thomas J Bonitz, Christine M Lomiguen, Justin Chin

**Affiliations:** 1 Medical Education, Lake Erie College of Osteopathic Medicine, Erie, USA; 2 Family Medicine, Millcreek Community Hospital, Erie, USA; 3 Pathology, Lake Erie College of Osteopathic Medicine, New York, USA; 4 Family Medicine, LifeLong Medical Care, Richmond, USA

**Keywords:** supraspinatus, rotator cuff repair surgery, non-opiate pain control, counterstrain, muscle energy, osteopathic manipulative medicine, chronic shoulder pain, rotator cuff tear management, rotator cuff pathology, rotator cuff tears

## Abstract

Rotator cuff tears, particularly involving the supraspinatus muscle and/or tendon, are highly prevalent among individuals engaged in repetitive shoulder motions. Occupations demanding constant and repetitive shoulder movements are especially susceptible to rotator cuff injuries, potentially leading to prolonged joint wear and tear and an increased likelihood of joint replacement. Considering the impact of social determinants of health, including access to healthcare and socioeconomic status, it is imperative to explore conservative treatment modalities that alleviate financial burdens and reduce lengthy recovery periods. In this report, we present a case of a 64-year-old female hairdresser diagnosed with a chronic partial thickness rotator cuff tear who remained unresponsive to physical therapy and chiropractic manipulation but exhibited improvement following osteopathic manipulative treatment. Additionally, osteopathic considerations and pertinent literature are reviewed to provide insight into the broader context of shoulder pain management.

## Introduction

Rotator cuff tears can elicit considerable pain and impede shoulder function, significantly impacting an individual's daily activities and overall quality of life. In the United States, rotator cuff tears are observed in approximately 10% of patients aged 60 and above and can encompass a spectrum of presentations, ranging from asymptomatic incidentalomas to severely debilitating shoulder pain and pathological conditions [1]. Rotator cuff tears can involve one or all of the following muscles: the supraspinatus, infraspinatus, teres minor, and subscapularis muscles, with the supraspinatus tendon being the most commonly affected structure [2]. Typical signs and symptoms include pain, weakness, crepitus, and altered sensation, often elicited through provocative maneuvers such as Codman's (drop arm) and Jobe's (empty can) tests [3]. Management of rotator cuff tears varies based on their severity, with conservative approaches, including rest, ice, physical therapy, and anti-inflammatory medications, typically prescribed for mild to moderate cases. These treatments aim to alleviate pain and enhance shoulder function by fortifying the surrounding musculature [4].

For more severe instances or when conservative measures prove ineffective, surgical intervention may be recommended, involving the repair and/or reattachment of affected muscles or tendons [5]. Recovery time is contingent upon the selected surgical approach and underlying comorbidities, ranging from approximately six weeks for initial healing to up to nine months for a full return to strength. Post-surgery rehabilitation is crucial, as physical therapy has demonstrated efficacy in restoring shoulder strength, mobility, and function [6]. Nonetheless, it is imperative to consider that this timeline may not adequately account for social determinants of health and financial or socioeconomic factors, particularly concerning the time off work required for recovery. Consequently, osteopathic manipulative medicine (OMM) can serve as an adjunctive measure to conservative treatment or surgical intervention, targeting associated somatic dysfunctions and fostering homeostasis to promote healing [7-11].

In this context, here we present a case of chronic rotator cuff tear in a 64-year-old Caucasian woman, who exhibited inadequate response to conservative management and faced financial barriers hindering surgical intervention. Notably, the patient experienced improvement following OMM treatments in which exploration of shoulder pathology from an osteopathic standpoint is reviewed as well as its implications for patient care.

## Case presentation

**Figure 1:**
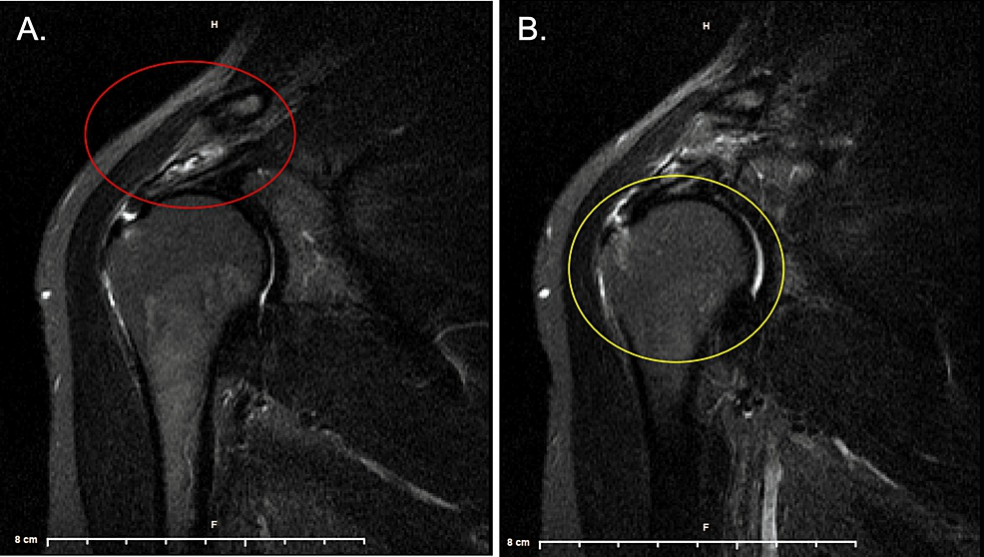
Magnetic resonance imaging of the right shoulder in coronal view with two millimeter partial tear in the distal supraspinatus tendon (A: red circle) along with subacromial-subdeltoid fluid in space (B: yellow circle).

A 64-year-old right-handed female with a three-year history of chronic right shoulder pain and limited range of motion, presented to outpatient primary care for evaluation. She had a past medical history of hypertension, migraines, hiatal hernia, and gastroesophageal reflux disease, and did not have any significant surgical or family history. Social history was significant for occupational exposure to repetitive overhead and shoulder motions as a self-employed hairdresser, working six to nine-hour shifts, five days a week. Two years prior, she was evaluated by orthopedic surgery in which magnetic resonance imaging showed a two-millimeter partial tear of the distal supraspinatus tendon (Figure 1). She was offered rotator cuff surgery and repair; however, she declined due to employment constraints, primarily being time off work along with cost. The patient proceeded with non-surgical management, but experienced limited relief after six months of chiropractic adjustments, along with failed improvement with over-the-counter pain medications, heat, ice, physical therapy, and other modalities.

Vitals were within normal range. Physical exam revealed reduced shoulder flexion between 15-90 degrees on the right, however, was ultimately able to maintain shoulder flexion at 90 degrees with the assistance of her other arm, which was a coping mechanism that the patient created to keep up with her hairdressing work. In the right arm, she reported pain at the greater and lesser tubercles of the humerus, with the worst subjective pain experienced at the deltoid tuberosity during abduction. Muscle strength was 5/5 in the left upper extremity and bilateral lower extremities. In the right upper extremity, initial abduction strength was 1/5, with improvement to 5/5 after 75 degrees of abduction. The patient denied any other muscle weakness, abnormal sensation, or pain in locations other than her right shoulder. Special tests for rotator cuff and shoulder pathology were positive in the drop arm and empty can test, while O'Briens, Hawkins, and cross-body adduction tests were negative.

**Figure 2:**
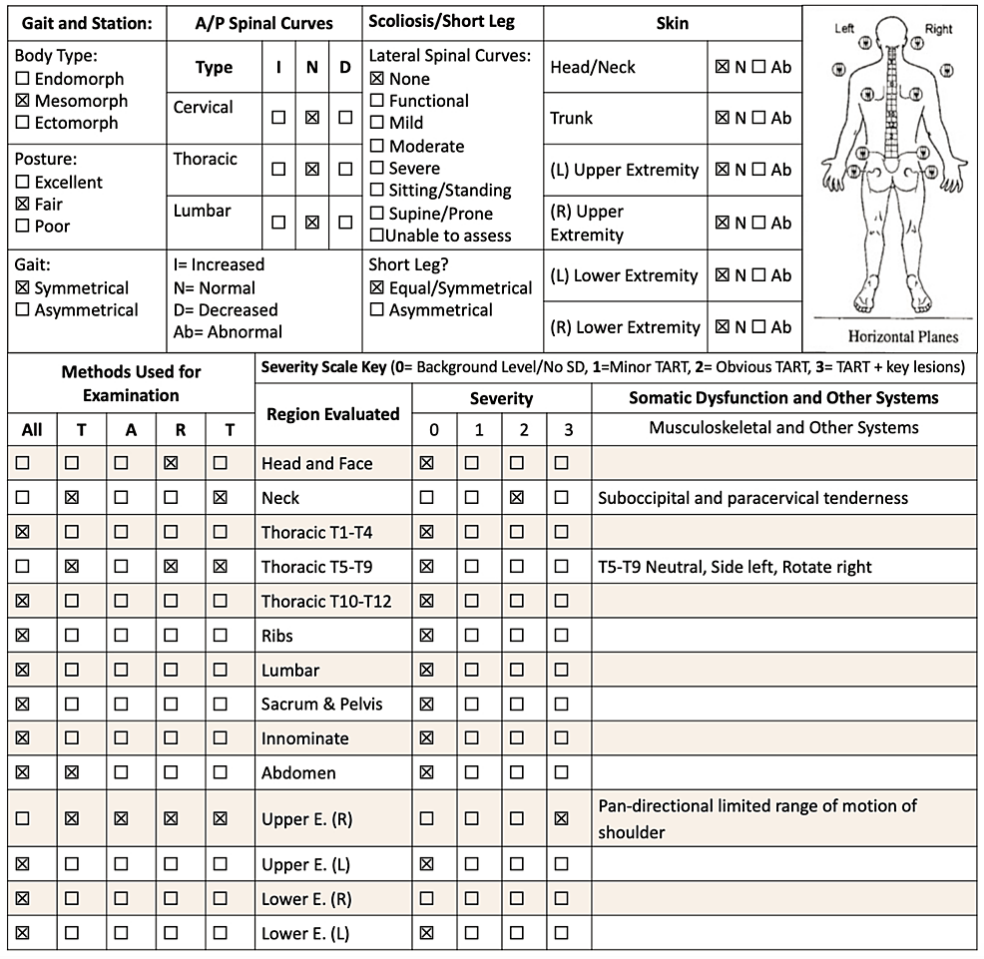
Complete osteopathic physical exam with significant findings. Originally adapted from the Journal of the American Osteopathic Association with significant findings.

An osteopathic structural exam revealed hypertonic and tender trapezius on the right on palpation as well as an exquisitely tender bicipital point. Complete findings are detailed in Figure 2.

Osteopathic manipulative treatment was offered to address the somatic dysfunctions found in Figure 2, with the initial treatment comprising of myofascial release and high-velocity-low amplitude manipulation of the thoracic spine providing substantial relief. Additional treatments targeted surrounding somatic dysfunctions included but were not limited to direct inhibition and muscle energy to the trapezius muscles, counter strain to the biceps brachii and supraspinatus, and facilitated the positional release and Still technique for the first rib. Biweekly treatments occurred for the first month followed by maintenance treatment weekly, with a complete return of range of motion and pain relief four months afterward.

Currently, the patient is two years of status post initial orthopedic assessment and subsequent osteopathic treatment undergoes monthly maintenance treatment with only mild residual pain, and has retained a complete range of motion.

## Discussion

Rotator cuff tendinopathy is the most common cause of shoulder pain, especially among patients who have occupations that require repetitive motion of the shoulder [12]. In addition to repetitive motion, it has been shown that overhead action and progressive use throughout the day can exacerbate pain and weakness associated with rotator cuff tears [13]. MRI can be employed as a diagnostic study if conservative management proves ineffective, however emerging studies have focused on ultrasound as a diagnostic method in acute tendinopathy [14]. Imaging may not necessarily predict outcomes, especially when socioeconomic or other factors play into management [15]. The presented case illustrates the potential impact of rotator cuff tendinopathy on hairdressers, whose occupation entails prolonged arm elevation, leading to muscle irritation. By balancing and reducing the hypertonicity in the patient's shoulder girdle, significant pain reduction was achieved. It is noteworthy that some rotator cuff tendinopathies may remain asymptomatic, while others, as observed in this case, can be profoundly debilitating [12, 13]. The variability in the patient presentation of rotator cuff tendinopathies poses challenges in devising a generalized treatment plan applicable to all scenarios.

Somatic dysfunction is defined as impaired components of the somatic (body framework) system and can include the musculoskeletal, nervous, and lymphatic systems. In osteopathic medicine, its core tenets recognize that “rational treatment is based upon an understanding of the basic principles of body unity, self-regulation, and the interrelationship of structure and function”[7]. Therefore, the osteopathic manipulative treatment regimen utilized these tenets to address somatic dysfunction as the direct and indirect modalities of muscle energy and counter strain engaged the Golgi tendon reflex, which inhibits hypertonic muscles, promoting relaxation and correction of the dysfunction [16]. In addition, other osteopathic techniques were implemented, each with its own theorized mechanisms for restoring homeostasis. In the case presentation, the patient benefited from these treatments with symptomatic relief.

Studies have demonstrated that patients with rotator cuff tendinopathy benefit from osteopathic manipulative treatment, particularly in pain reduction and the need for further intervention [17]. While focus is typically placed on acute rotator cuff tendinopathy, further research is needed to better understand and develop non-surgical long-term management with an emphasis on osteopathic protocols. Osteopathic research, incorporating double-blinded studies, is warranted to ascertain the optimal treatment approach for rotator cuff tears in non-surgical candidates. The design and execution of such studies are inherently complex due to standard barriers associated with randomized-controlled trials as well as the additional challenges associated with osteopathic research which include the necessity of devising and implementing "sham therapy" to blind the operator [18]. Despite these challenges, this case report highlights the importance of continued research, especially in elucidating the specific mechanisms that can create optimal efficacy in shoulder pain management.

## Conclusions

Chronic rotator cuff tears may benefit from osteopathic manipulative treatment. As seen in this case, the patient was able to resume occupational activities with diminished restrictions and alleviated pain. In the context of managing patients with shoulder pain, osteopathic healthcare providers should contemplate integrating osteopathic treatment into their therapeutic protocols. Furthermore, non-osteopathic healthcare practitioners should consider the referral of patients to osteopathic manipulative medicine specialists as a viable non-invasive alternative for managing shoulder pain. Further studies are needed in the future to validate osteopathic treatment for chronic rotator cuff tears.
